# A novel hybrid CLARA and fuzzy time series Markov chain model for predicting air pollution in Jakarta

**DOI:** 10.1016/j.mex.2025.103202

**Published:** 2025-02-05

**Authors:** Nurtiti Sunusi, Ankaz As Sikib, Sumanta Pasari

**Affiliations:** a Stochastic Modeling Research Group, Department of Statistics, Faculty of Mathematics and Natural Sciences, Hasanuddin University, Jl. Perintis Kemerdekaan Km. 10, Makassar 90245, Indonesia; b Department of Mathematics, Birla Institute of Technology and Science, Pilani, India

**Keywords:** Air pollution, CLARA, Clustering, Forecasting, Hybrid clustering fuzzy time series, Hybrid Clustering Large Applications and Fuzzy Time Series Markov Chain

## Abstract

Air pollution poses a significant challenge to public health and the global environment. The Industrial Revolution, advancing technology and society, led to elevated air pollution levels, contributing to acid rain, smog, ozone depletion, and global warming. Poor air quality increases risks of respiratory inflammation, tuberculosis, asthma, chronic obstructive pulmonary disease (COPD), pneumoconiosis, and lung cancer.

In this context, developing reliable air pollution forecasting models is imperative for guiding effective mitigation strategies and policy interventions. This study presents a daily air pollution prediction model focusing on Jakarta's sulfur dioxide (SO₂) and carbon monoxide (CO) levels, leveraging a hybrid methodology that integrates Clustering Large Applications (CLARA) with the Fuzzy Time Series Markov Chain (FTSMC) approach.

The analysis revealed five distinct clusters, with medoid selection refined iteratively to ensure stabilization. A 5 × 5 Markov transition probability matrix was subsequently constructed for modeling the data. Predicted values for SO₂ and CO in Jakarta using the CLARA-FTSMC hybrid method showed strong alignment with the actual data. Forecasting accuracy results for SO₂ and CO in Jakarta, based on Mean Absolute Error (MAE) and Root Mean Square Error (RMSE), showed excellent performance, underscoring the efficacy of the CLARA-FTSMC hybrid approach in predicting air pollution levels.

• The CLARA-FTSMC hybrid method demonstrates high effectiveness in analyzing large datasets, addressing the limitations of previous hybrid clustering fuzzy time series methods.
• The number of fuzzy time series partitions is optimally determined based on clustering results obtained through the gap statistic approach, ensuring robust partitioning.
• The forecasting accuracy of the CLARA-FTSMC hybrid method, evaluated using MAE and RMSE, showed excellent performance in predicting daily air pollution levels of SO₂ and CO in Jakarta.

## Graphical abstract

**Figure:**
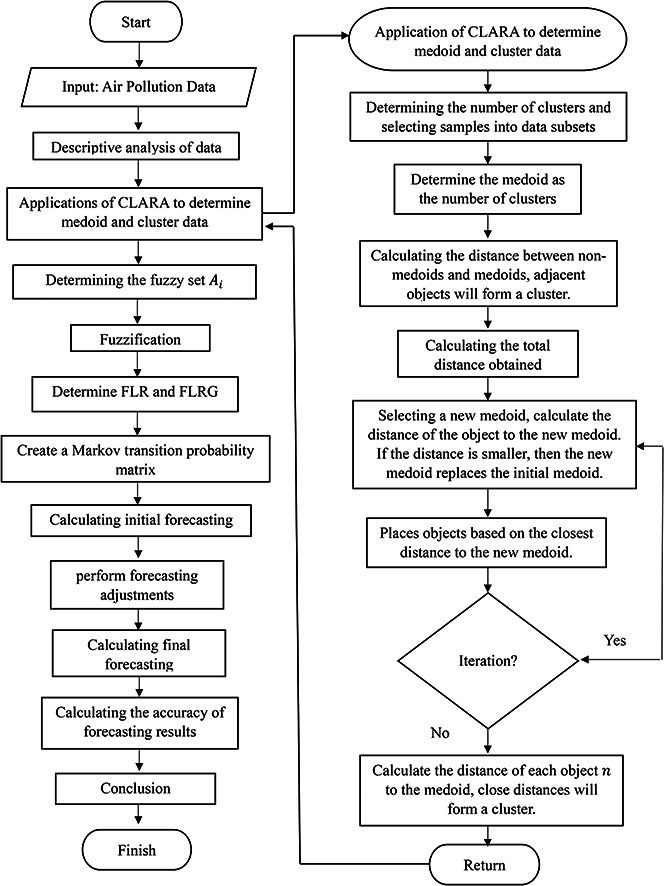

Specifications table

**Table:** 

Subject area:	Mathematics and Statistics
More specific subject area:	Statistics; Hybrid Clustering Fuzzy Time Series; Air Pollution
Name of your method:	Hybrid Clustering Large Applications and Fuzzy Time Series Markov Chain
Name and reference of original method:	Finding Groups in Data: An Introduction to Cluster Analysis (1991), Gentle JE, Kaufman L, Rousseuw PJ, Biometrics, Vol. 47, 788 p. A fuzzy time series-Markov chain model with an application to forecast the exchange rate between the Taiwan and us Dollar (2012), Tsaur RC, Int J Innov Comput Inf Control, 8(7 B):4931–42.
Resource availability:	The data utilized in this study were sourced from the official website of the DKI Jakarta Environment Agency (https://lingkunganhidup.jakarta.go.id/). The dataset comprises daily air pollution standard index records spanning from January 2021 to August 31, 2024, ensuring comprehensive coverage for the analysis period.

## Background

Air pollution remains one of the most pressing challenges for public health and the global environment. Since the Industrial Revolution, significant advancements in technology, energy, and societal development have provided immense benefits to humanity. However, these advancements have also resulted in severe environmental consequences, particularly the escalation of air pollution. Air pollution contributes to numerous environmental issues, including acid rain, smog, ozone depletion, and global warming [1]. Moreover, poor air quality is strongly associated with various health risks, such as respiratory inflammation, tuberculosis, asthma, chronic obstructive pulmonary disease (COPD), pneumoconiosis, and lung cancer [2,3].

Air pollution is defined as the alteration of air composition due to the presence of hazardous substances, including particulate matter (PM), sulfur dioxide (SO₂), carbon monoxide (CO), nitrogen dioxide (NO₂), and other heavy metals. According to the World Air Quality Report (2023), Indonesia ranks first as the most polluted country in Southeast Asia [4]. As of June 23, 2024, Jakarta recorded the second-highest air pollution levels globally, with an Air Quality Index (AQI) of 160 [5]. The primary drivers of air pollution in Jakarta include industrialization, fossil fuel combustion, mining activities, and the annual 10% increase in private vehicle ownership [6,7].

Although the Jakarta government has implemented various policies, such as promoting public transportation, deploying air quality task forces, and imposing disincentives for parking fees, air quality continues to deteriorate. Therefore, developing accurate air pollution forecasting models is increasingly critical to support the formulation of effective mitigation policies.

One interesting approach in time series analysis is the use of fuzzy logic-based methods. Fuzzy logic has been proven to provide more effective results in solving various practical problems, including the forecasting of time series data [8]. Fuzzy logic allows us to incorporate uncertainty in time series data, which is often caused by variations and external factors that are difficult to consider by classical analysis methods [9].

Fuzzy time series (FTS) was introduced by Song and Chissom [10] to predict enrollments at the University of Alabama. Since then, various FTS methods have been developed such as weighted [11], Chen [12], Markov [13] and multiple attributes [14]. One of the recent methods that has received attention in fuzzy-based time series analysis is the fuzzy time series Markov chain (FTSMC). FTSMC is an approach that combines fuzzy logic concepts with chain models to forecast future values based on fuzzy partitions of time series data. The partitioning allows various states to occur in the time series, whereas the fuzzy concept allows us to measure the degree of membership of each state in each partition.

Based on the studies conducted by [15,16], FTSMC is the most preferable method based on MSE and MAPE metrics in compared to other FTS methods. However, FTS has some issues, such as determining the exact number of partitions; also, the length of the interval does not have a definite formula in its calculation [17]. The relationship between the number of partitions and the interval length has been addressed in previous research works [15,18]. In fact, the number of partitions and the length of the interval greatly affect the formation of the membership relationship (FLR), resulting in differences in the accuracy of the forecasting results.

Therefore, the selection of the optimal number of partitions is an interesting problem that needs to be discussed. Some previous studies have tried to incorporate clustering methods to optimize the partitions in the FTS method [[19], [20], [21], [22]]. However, in determining the optimal partitioning, *k*-means and *k*-medoid methods still have shortcomings, as they are less effective in analyzing large data compared to improved methods such as clustering large applications (CLARA).

The CLARA is robust to large amounts of data and can cope with outliers [20]. Thus, the incorporation of CLARA in the FTSMC analysis stage results in a very flexible method in forecasting large amounts of daily air pollution data. This research aims to develop a prediction model for sulfur dioxide (SO₂) and carbon monoxide (CO) based daily air pollution in Jakarta, using a hybrid approach of Clustering Large Applications (CLARA) and Fuzzy Time Series Markov Chain (FTSMC). The model is expected to provide more accurate projections to support strategic decision-making in urban air pollution mitigation.

## Method details

### Fuzzy Time Series

The Fuzzy Time Series (FTS) method typically utilizes historical data in linguistic form [21]. The FTS process consists of defining the universe of discourseU, partitioningUinto several intervals, fuzzification, establishing fuzzy relationships, and defuzzification.

**Definition 1**

Let$\;U=\{u_{1},\;u_{2},\;u_{3},\;\ldots ,\;u_{n}\}\;$be the universe of discourse, where$\;u_{n}\;\left(i=1,\ldots ,\;n\right)\;$represents possible linguistic values withinU. The linguistic fuzzy variableAorUis defined as:

(1) $$A_{i}=\frac{f_{A_{i}}\left(u_{1}\right)}{u_{1}}+\frac{f_{A_{i}}\left(u_{2}\right)}{u_{2}}+\ldots +\frac{f_{A_{i}}\left(u_{n}\right)}{u_{n}}$$

Where$\;{f_{A}}_{i}\;$is the membership function of fuzzy set$\;{f_{A}}_{i}:U\to \left[0,1\right],\;\;{f_{A}}_{i}\left(u_{r}\right)\in \left[0,1\right]\;$and1<r<n.

**Definition 2**

LetY(t)(t=1,2,...,n),be a real-valued time series, whereY(t)is defined over the fuzzy set$\;f_{i}\left(t\right),\;i=1,2,3,\ldots ,n$. ThenF(t)represents the fuzzy time series ofY(t).

**Definition 3**

If$\;Y\left(t\right)=A_{j}\;$is caused by$\;Y\left(t-1\right)\;=\;A_{i},\;$then the fuzzy logical relationship (FLR) is expressed as:$\;A_{i}\to \;A_{j}$.

**Definition 4**

If an FLR originates from state$\;A_{2}$, and transitions to other states$\;A_{j}$, (j=1,2,3,…n), such as$\;A_{2}\to A_{3},\;A_{2}\to A_{2},\;A_{2}\to A_{1},\;$the FLRs are grouped into a fuzzy logical relationship group (FLRG) as follows:

(2) $$A_{2}\to A_{1},A_{2},A_{3}$$

Fuzzification transforms numerical data into linguistic values, forming the FLR. This step requires determining the upper and lower bounds using the following equations:

(3) $$\;ub_{i}=\frac{cluster\;center_{i}+cluster\;center_{i+1}}{2}$$

Here$\;i=1,\;2,\;\ldots ,\;k;\;ub_{i}\;$is the upper boundary of thei-th interval, while the lower boundary of the next interval is$\;lb_{i+1}.\;$For the first and last clusters, where no prior or subsequent centers exist, the lower bound$\;lb_{1}\;$and upper bound$\;ub_{k}\;$are computed as:

(4) $$ub_{k}=\;cluster\;center_{k}+\left|max_{data}-cluster\;center_{k}\right|$$

(5) $$lb_{1}=cluster\;center_{1}-\left|cluster\;center_{1}-\;min_{data}\right|$$

[23,24]

### Fuzzy time series Markov chain

The transition probability matrix in Markov chain is constructed as a(p×p) matrix, whereprepresents the number of fuzzy sets [22]. The equation to determine the transition probability between states is as follows:

(6) $$P_{ij}=\frac{r_{ij}}{r_{i}}$$

Where:

$P_{ij}$: Transition probability from state$\;A_{i}\;$to$\;A_{j}$

$r_{ij}$: Number of transitions from state$\;A_{i}\;$ke$\;A_{j}$

$r_{i}$: Total number of data points in state$\;A_{i}$

The transition probability matrix **P** can be expressed as:

$$P\;=\;\left[\begin{array}{cc} \begin{array}{cc} P_{11} & P_{12}\\ P_{21} & P_{22} \end{array} & \begin{array}{cc} \ldots & P_{1p}\\ \ldots & P_{2p} \end{array}\\ \begin{array}{cc} \vdots & \vdots \\ P_{p1} & P_{p2} \end{array} & \begin{array}{cc} \ddots & \vdots \\ \ldots & P_{pp} \end{array} \end{array}\right]$$

1) The initial forecast values are determined using the following rules:

**Table:** 

Rule 1	If a fuzzy set does not have a Fuzzy Logical Relationship (FLR), ($A_{i}\to \;\emptyset$), andY(t−1)at time t−1falls into$\;A_{i}$, then the forecast value$\;F_{t}\;$is$\;m_{i}$_,_ where$\;m_{i}\;$is the midpoint of interval$\;x_{i}$.
Rule 2.	If the Fuzzy Logical Relationship Group (FLRG)$\;A_{i}\;$represents a one-to-one relationship ($A_{i}\to \;A_{q}$), andY(t−1)at timet−1falls into state$\;A_{i}$, then the forecast value$\;F_{t}\;$is m_q_, where m_q_ is the midpoint of$\;x_{q}\;$in the FLRG formed at timet−1.
Rule 3.	If the FLRG$\;A_{i}\;$represents a one-to-many relationship ($A_{j}\to \;A_{1},\;A_{2}$,$\;A_{3},...,A_{q}$, j = 1, 2, 3, …, q), andY(t−1)at timet−1falls into state$\;A_{j}$, the forecastF(t)is calculated as:
	$F\left(t\right)=m_{1}P_{i1}+m_{2}P_{i2}+\cdots +m_{i-1}P_{i(i-1)}+Y\left(t\right)P_{ii}\;$+$\;m_{i+1}+1P_{i(i+1)}+\cdots +m_{n}P_{in}\;\;\;\;\;\;\;\;\;\;\;\;\;\;\;\;\;\;\left(7\right)$
	where,$\;m_{1},\;m_{2},\;\ldots ,\;m_{n}\;$are the midpoints of$\;u_{1},\;u_{2},\ldots ,\;u_{n}\;$ and $\;m_{i}\;$is replaced withY(t)for state$\;A_{i}\;$to improve forecasting accuracy.

2) To improve the forecast accuracy, an adjustment is made by adding the difference between the actual valueY(t),and the previous value, as follows:
  (8) $$\hat{F}\left(t+1\right)=\;F\left(t+1\right)+diff\;\left(Y\left(t\right)\right)$$

diff(Y(t)) is the difference between the actual value(Y(t))at timetand the previous actual valueY(t−1).

(9) $$diff\left(Y\left(t\right)\right)=\;\left\{ \begin{array}{c} 0,\;if\;Y\left(t=1\right)\\ Y\left(t\right)-Y\left(t-1\right),\;if\;Y\left(t\geq 2\right) \end{array}\right.$$

where:

**Table:** 

Y(t)	: Actual data at periodt
F(t)	: Initial forecast result at periodt
$\hat{F}\left(t\right)$	: Adjusted forecast result at periodt

### Euclidean distance

Euclidean distance is a method for calculating the distance between points in Euclidean space, which is subsequently used to group these points into clusters based on their proximity [25].

(10) $$d\left(x,y\right)=\sqrt{\sum_{i=1}^{n}{\left(x_{k}-y_{i}\right)}^{2}\;},k=1,\;2,\ldots ,\;c$$

where:

d(x,y): Euclidean distance between$\;x_{k}\;$and$\;y_{i}$$x_{k}$: thek-th cluster center value

$y_{i}$: thei-th actual data value(i=1,2,…,n)

### Gap statistics

The Gap Statistics method is used to determine the optimal number of clusters. It achieves this by comparing the intra-cluster variation of the actual data with the expected variation from randomly generated data. The Gap Statistics method demonstrates higher accuracy when integrated with the CLARA algorithm, which efficiently incorporates large datasets through sampling. The Gap Statistic value is calculated using the following equation:

(11) $$Gap\left(k\right)=\frac{1}{B}\sum_{b=1}^{B}\log\left(W_{kb}\right)-log\left(W_{k}\right)$$

where:

**Table:** 

Gap(k)	: The gap statistic for the optimal number of clustersk
B	: The number of bootstrap samples used in the gap statistic method
$W_{kb}$	: The intra-cluster dispersion forkcluster in the b^th^ bootstrap sample
$W_{k}$	: The within-cluster variation forkcluster in the original dataset

### Clustering large applications

Clustering Large Applications (CLARA) utilizes medoids as cluster centers to group large-scale data and is robust against outliers [26]. CLARA divides large datasets into smaller subsets while ensuring optimal medoid selection. The sample size for each subset is determined using the following equation:

(12) min(40+(2×K))

where:

**Table:** 

K	: Number of clusters.

The fundamental principle of the partition around medoids (PAM) algorithm is to minimize the dissimilarity between objects within a cluster by iteratively swapping the medoid and non-medoid objects until convergence [27]. Typically, the process of finding a new medoid is repeated to achieve the best medoid with the smallest total distance representing the cluster. The formula for evaluating the medoid swap is:

(13) S=TotalEuclideandistanceofthenewmedoid−TotalEuclideandistanceoftheoldmedoid

Where:

**Table:** 

If *S* < 0	the medoid swap is repeated
If *S* > 0	the iteration stops

### Accuracy of the forecasting model

The accuracy of a forecasting model improves as the error value decreases. A lower error indicates higher accuracy and vice versa [28,29]. The formula to measure the accuracy of time series analysis results is as follows (Table 1):

(14) $$MAE\;=\;\frac{\sum_{t=1}^{T}|y_{t}-{\hat{F}}_{t}|}{n}$$

(15) $$RMSE\;=\;\sqrt{\frac{\sum_{t=1}^{T}{\left(y_{t}-{\hat{F}}_{t}\right)}^{2}}{n}}$$

**Table 1:** Model forecast accuracy criteria.

**Accuracy Value**	**Criterion**
≤ 10 10 < Value ≤ 20 20 < Value ≤ 50 > 50	Excellent Good Fair Poor

### Method validation

In forecasting sulfur dioxide and carbon monoxide air pollution in Jakarta using the hybrid CLARA and FTSMC, we utilized secondary data, specifically the daily air quality index from January 2021 to August 31, 2024. The dataset includes two dependent variables. The details of these variables are provided in Table 2.

**Table 2:** Variable description.

**Variable**	**Description**
$y_{1}$	Daily sulfur dioxide air pollution data
$y_{2}$	Daily carbon monoxide air pollution data

### Descriptive analytics

**Table 3:** Descriptive Analysis of SO₂ and CO Pollution in Jakarta from January 1, 2021 to August 31, 2024.

	Minimum	Maximum	Mean
SO_2_	8	112	36,27
CO	3	55	15,12

#### Selection of optimal cluster number

**Fig. 1:**
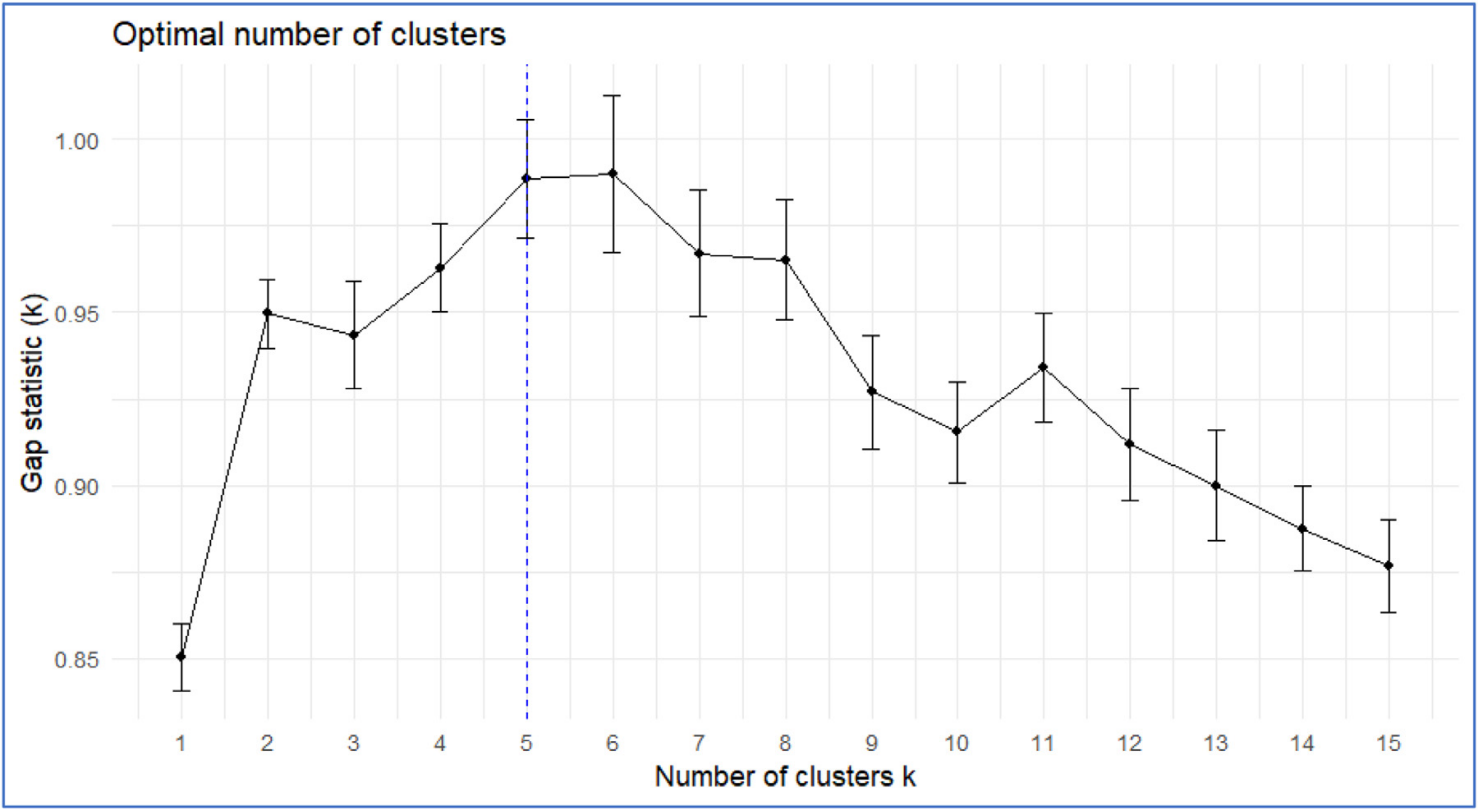
Determination of the optimal number of clusters using Gap Statistics, with a maximum of 15 clusters and bootstrapping of 100 iterations.

Gap Statistic is particularly effective in the CLARA algorithm, which works with large datasets. It determines the optimal number of clusters by comparing the clustering results with expectations derived from random data, ensuring more accurate clustering outcomes (Fig. 1).

#### Clustering large applications analysis

Based on Eq. (12), the number of samples from the actual data is determined in the process of selecting the optimal medoid (Tables 4, Table 6, Table 7, Table 8, Table 9).

(40+(2x5))=50

**Table 4:** Samples of the CLARA algorithm in selecting the initial medoids, where each sample represents a data sequence from the actual SO_2_ and CO air pollution datasets.

[1]	19	21	48	77	92	125	150	178	219	270
[11]	274	287	290	352	364	368	369	382	412	418
[21]	465	529	578	712	766	802	809	863	867	899
[31]	918	928	968	995	1022	1026	1028	1064	1104	1116
[41]	1142	1153	1162	1175	1196	1208	1220	1233	1234	1332

#### Selection of the new medoid

##### Comparison of initial and new medoid total Euclidean distance

The total Euclidean distance of the initial medoid is 8.850,34, while the total Euclidean distance of the new medoid is 9.531,82 using Eq. (13), the result is:

S=8.850,34−9.531,82=681,48

Since(S)>0, the iteration stops, and the initial medoid becomes the final medoid for the air pollution data of SO₂ and CO in Jakarta.

### Medoid intervals

The medoid results from Table 5 serve as the basis for forming the discourse interval universeUusing Eqs. (3)–(5) (Table 10).

**Table 5:** Initial medoids based on the sample data of SO₂ and CO air pollution.

Cluster	Medoid		Label
	SO₂	CO	
1	18	9	Very Low
2	30	11	Low
3	44	12	Moderate
4	46	20	High
5	50	22	Very High

**Table 6:** Distance calculation between objects and initial medoids for SO₂ and CO air pollution data.

N	Date	SO₂	CO	Cost1	Cost2	Cost3	Cost4	Cost5	Proximity
1	01/01/2021	29	6	11,40	5,10	16,16	22,02	26,40	5,10
2	02/01/2021	27	7	9,22	5,00	17,72	23,02	27,46	5,00
3	03/01/2021	25	7	7,28	6,40	19,65	24,70	29,15	6,40
4	04/01/2021	24	4	7,81	9,22	21,54	27,20	31,62	7,81
⋮	⋮	⋮	⋮	⋮	⋮	⋮	⋮		⋮
1336	28/08/2024	14	24	15,52	20,62	32,31	32,25	36,06	15,52
1337	29/09/2024	14	26	17,46	21,93	33,11	32,56	36,22	17,46
1338	30/08/2024	15	28	19,24	21,67	33,12	32,02	35,51	19,24
1339	31/08/2024	14	25	16,49	21,26	32,70	32,39	36,12	16,49
						Cost Total			8.850,34

**Table 7:** Samples of CLARA algorithm for new medoid selection, where each sample represents a data sequence on actual air pollution data for SO₂ and CO.

[1]	44	80	86	87	89	100	110	119	141	186
[11]	188	224	235	335	343	377	396	397	420	434
[21]	450	463	503	533	566	574	576	595	666	698
[31]	737	779	809	866	867	892	936	943	1032	1075
[41]	1102	1115	1121	1129	1178	1180	1205	1254	1332	1336

**Table 8:** New medoids based on actual SO₂ and CO air pollution data samples.

Cluster	Medoid		Label
	SO₂	CO	
1	14	11	Very Low
2	25	12	Low
3	42	15	Moderate
4	49	24	High
5	51	25	Very High

**Table 9:** Calculation of object distances to new medoids for SO2 and CO air pollution data.

N	Date	SO₂	CO	Cost1	Cost2	Cost3	Cost4	Cost5	Proximity
1	01/01/2021	29	6	15,81	7,21	15,81	26,91	29,07	7,21
2	02/01/2021	27	7	13,60	5,39	17,00	27,80	30,00	5,39
3	03/01/2021	25	7	11,70	5,00	18,79	29,41	31,62	5,00
4	04/01/2021	24	4	12,21	8,06	21,10	32,02	34,21	8,06
⋮	⋮	⋮	⋮	⋮	⋮	⋮	⋮		⋮
1336	28/08/2024	14	24	13,00	16,28	29,41	35,00	37,01	13,00
1337	29/09/2024	14	26	15,00	17,80	30,08	35,06	37,01	15,00
1338	30/08/2024	15	28	17,03	18,87	29,97	34,23	36,12	17,03
1339	31/08/2024	14	25	14,00	17,03	29,73	35,01	37,00	14,00
						**Cost Total**			**9.531,82**

**Table 10:** Medoid intervals based on medoids in Table 5 for SO₂ and CO air pollution data.

SO₂		CO	
Interval	Midpoint	Interval	Midpoint
$u_{1}=\left[08,0;\;24,0\right)$	$m_{1}$= 16,0	$u_{1}=\left[03,0;\;10,0\right)$	$m_{1}$= 6,5
$u_{2}=\left[24,0;\;37,0\right)$	$m_{2}$= 30,5	$u_{2}=\left[10,0;\;11,5\right)$	$m_{2}$= 10,75
$u_{3}=\left[37,0;\;45,0\right)$	$m_{3}$= 41,0	$u_{3}=\left[11,5;\;16,0\right)$	$m_{3}$= 13,75
$u_{4}=\left[45,0;\;71,0\right)$	$m_{4}$= 58,0	$u_{4}=\left[16,0;\;21,0\right)$	$m_{4}$= 18,5
$u_{5}=\left[71,0;\;112,0\right)$	$m_{5}$= 91,5	$u_{5}=\left[21,0;\;55,0\right)$	$m_{5}$= 38,0

## Fuzzy time series Markov chain

### Fuzzification, fuzzy logic relationship (FLR), and fuzzy logic relationship group (FLRG)

Fuzzy Logic Relationships (FLR) is a concept in FTS that is used to capture the relationship between fuzzy sets in time series shown in Table 11. While FLRG is an accumulation of FLR between fuzzy sets to help understand historical data patterns for forecasting purposes as shown in Table 12.

**Table 11:** Fuzzification and FLR results for SO₂ and CO air pollution data.

N	Date	SO₂	Fuzzification	FLR	CO	Fuzzification	FLR
1	01/01/2021	29	$A_{2}$	−	6	$A_{1}$	−
2	02/01/2021	27	$A_{2}$	$A_{2}\to A_{2}$	7	$A_{1}$	$A_{1}\to A_{1}$
3	03/01/2021	25	$A_{2}$	$A_{2}\to A_{2}$	7	$A_{1}$	$A_{1}\to A_{1}$
4	04/01/2021	24	$A_{2}$	$A_{2}\to A_{2}$	4	$A_{1}$	$A_{1}\to A_{1}$
⋮	⋮	⋮	⋮	⋮	⋮	⋮	⋮
1336	28/08/2024	14	$A_{1}$	$A_{1}\to A_{1}$	24	$A_{5}$	$A_{5}\to A_{5}$
1337	29/09/2024	14	$A_{1}$	$A_{1}\to A_{1}$	26	$A_{5}$	$A_{5}\to A_{5}$
1338	30/08/2024	15	$A_{1}$	$A_{1}\to A_{1}$	28	$A_{5}$	$A_{5}\to A_{5}$
1339	31/08/2024	14	$A_{1}$	$A_{1}\;\to A_{1}$	25	$A_{5}$	$A_{5}\to A_{5}$

**Table 12:** FLRG results for SO₂ and CO air pollution data.

**K**	**FLRG**	
	**SO₂**	**CO**
1	$A_{1}\;\to \;$(276)$\;A_{1}$, (27)$\;A_{2}.$	$A_{1}\;\to \;$(137)$\;A_{1}$, (42)$\;A_{2},\;$(29)$\;A_{3}$, (11)$\;A_{4},\;$(2)$\;A_{5}.$
2	$A_{2}\;\to \;$(27)$\;A_{1}$, (218)$\;A_{2},\;$(11)$\;A_{3}$, (5)$\;A_{4}.$	$A_{2}\;\to \;$(53)$\;A_{1}$, (94)$\;A_{2},\;$(81)$\;A_{3}$, (14)$\;A_{4},\;$(2)$\;A_{5}.$
3	$A_{3}\;\to \;$(1)$\;A_{1}$, (13)$\;A_{2},\;$(217)$\;A_{3}$, (49)$\;A_{4}.$	$A_{3}\;\to \;$(25)$\;A_{1}$, (87)$\;A_{2},\;$(156)$\;A_{3}$, (79)$\;A_{4},\;$(28)$\;A_{5}.$
4	$A_{4}\;\to \;$(2)$\;A_{2},\;$(52)$\;A_{3}$, (438)$\;A_{4},\;$(1)$\;A_{5}.$	$A_{4}\;\to \;$(5)$\;A_{1}$, (17)$\;A_{2},\;$(74)$\;A_{3}$, (90)$\;A_{4},\;$(58)$\;A_{5}.$
5	$A_{5}\;\to \;$(1)$\;A_{4}.$	$A_{5}\;\to \;$(4)$\;A_{2},\;$(35)$\;A_{3}$, (50)$\;A_{4},\;$(164)$\;A_{5}.$

### Transition probability matrix Markov

Based on the FLRG in Table 12, the next step is to form a 5 × 5 transition probability matrix based on Eq. (6). where$\;{W_{SO}}_{2}\;$is the transition probability matrix for sulfur dioxide and$\;W_{CO}\;$is the transition probability matrix for carbon monoxide.

$$W_{SO_{2}}=\left[\begin{array}{ccccc} 0,911 & 0,089 & 0,000 & 0,000 & 0,000\\ 0,103 & 0,835 & 0,042 & 0,019 & 0,000\\ 0,004 & 0,046 & 0,775 & 0,175 & 0,000\\ 0,000 & 0,004 & 0,105 & 0,888 & 0,002\\ 0,000 & 0,000 & 0,000 & 1,000 & 0,000 \end{array}\right]W_{CO}=\left[\begin{array}{ccccc} 0,620 & 0,190 & 0,131 & 0,050 & 0,009\\ 0,217 & 0,385 & 0,332 & 0,057 & 0,008\\ 0,067 & 0,232 & 0,416 & 0,211 & 0,075\\ 0,020 & 0,070 & 0,303 & 0,369 & 0,238\\ 0,000 & 0,016 & 0,138 & 0,198 & 0,648 \end{array}\right]$$

### Hybrid CLARA FTSMC forecasting results for SO₂ and CO air pollution

Defuzzification is performed in two stages: initial forecasting and adjustment of the forecast. The results of the defuzzification process are shown in Tables 13 **and** 14.

**Table 13:** Hybrid CLARA FTSMC forecasting results for SO₂ air pollution in Jakarta.

***N***	**Date**	**SO₂**	**Fuzzification**	**Initial Forecast**$\;(F_{t})$	**Final Forecast**$\;(\hat{F_{t}})$
1	01/01/2021	29	$A_{2}$	−	−
2	02/01/2021	27	$A_{2}$	56,45	49,45
3	03/01/2021	25	$A_{2}$	45,53	57,53
4	04/01/2021	24	$A_{2}$	58,89	48,89
⋮	⋮	⋮	⋮	⋮	⋮
1337	29/09/2024	14	$A_{1}$	15,47	15,47
1338	30/08/2024	15	$A_{1}$	15,47	15,47
1339	31/08/2024	14	$A_{1}$	15,47	16,47
1340	01/09/2024	-	-	-	16,47

**Table 14:** Forecast results for CO air pollution in Jakarta using Hybrid CLARA FTSMC.

**N**	**Date**	**CO**	**Fuzzification**	**Initial Forecast**$\;(F_{t})$	**Final Forecast**$\;(\hat{F_{t}})$
1	01/01/2021	6	$A_{1}$	−	−
2	02/01/2021	7	$A_{1}$	8,83	9,83
3	03/01/2021	7	$A_{1}$	9,45	9,45
4	04/01/2021	4	$A_{1}$	9,45	6,45
⋮	⋮	⋮	⋮	⋮	⋮
1337	29/09/2024	26	$A_{5}$	21,29	23,29
1338	30/08/2024	28	$A_{5}$	22,58	24,58
1339	31/08/2024	25	$A_{5}$	23,88	20,88
1340	01/09/2024	-	-	-	20,88

**Fig. 2:**
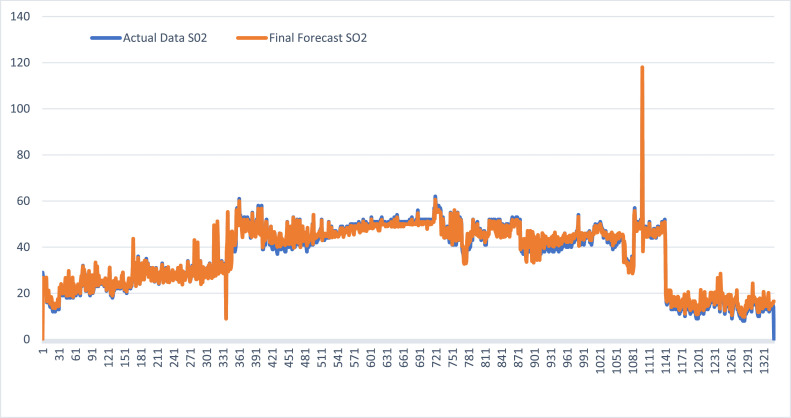
Graph of actual data and forecasted SO₂ values using Hybrid CLARA FTSMC.

The predicted value of SO₂ using the CLARA-FTSMC hybrid method has good agreement with actual data. The predicted value for September 1 was 16,47, while the actual value reported by the DKI Jakarta Environment Agency for the same day was 12. In addition, the forecasting accuracy assessed from the Mean Absolute Error (MAE) and Root Mean Square Error (RMSE) is classified as excellent (Fig. 2).

**Fig. 3:**
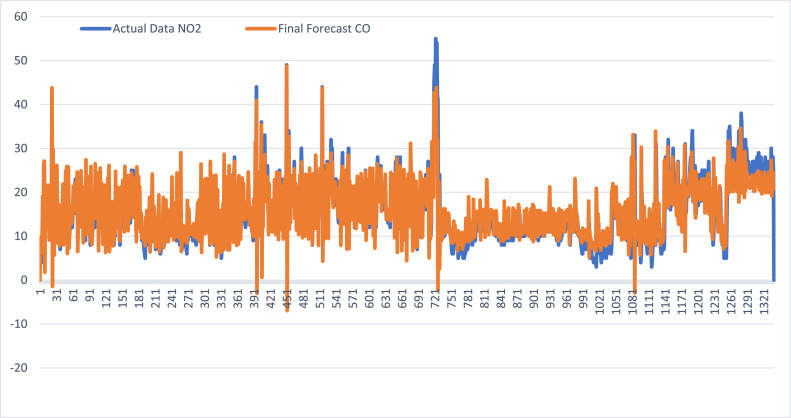
FTSMC Graph of actual data and forecasted CO values using Hybrid CLARA FTSMC.

The predicted value of CO using the CLARA-FTSMC hybrid method has good agreement with actual data. The predicted value for September 1 was 20,88, while the actual value reported by the DKI Jakarta Environment Agency for the same day was 22. In addition, the forecasting accuracy assessed from the Mean Absolute Error (MAE) and Root Mean Square Error (RMSE) is classified as excellent (Fig. 3 and Table 15).

**Table 15:** Forecast model accuracy for SO₂ and CO air pollution in Jakarta using Hybrid CLARA FTSMC.

**SO₂**		**CO**	
**MAE**	**RMSE**	**MAE**	RMSE
1,19	1,63	3,17	4,66

## Conclusion

Based on the descriptive analysis of daily air pollution data for SO₂ and CO in Jakarta (as shown in Table 2), SO₂ data from January 1, 2021 to August 31, 2024 has recorded an average value of 36,27, with a minimum value of 8 and a maximum of 112. Similarly, the CO data has showed an average value of 15,12, with a minimum value of 3 and a maximum of 55.

This research utilizes a hybrid methodology that combines Clustering Large Applications (CLARA) and Fuzzy Time Series Markov Chain (FTSMC). Statistical analysis of the gaps suggests the optimal number of clusters as five, with medoid selection completed at the initial optimal medoid point. This process has generated a 5 × 5 Markov transition probability matrix to effectively model the data.

Predicted values for SO₂ and CO using the CLARA-FTSMC hybrid method have showed strong alignment with the actual data. For September 1, the predicted value of SO₂ was 16,47, while the actual value reported by the DKI Jakarta Environment Agency was 12. Similarly, for CO, the predicted value was 20,88, as compared to the actual value of 22. In addition, the forecasting accuracy, evaluated by Mean Absolute Error (MAE) and Root Mean Square Error (RMSE), was classified as excellent.

## Limitations

1. Model prediction accuracy is assessed using Mean Absolute Error (MAE) and Root Mean Squared Error (RMSE).
2. This research focuses on a single area in Indonesia, specifically the air pollution forecasting in Jakarta, thus limiting the generalizability of the results to other areas that may have different environmental conditions.
3. The forecasting approach primarily relies on dependent variables and it has not incorporated independent variables to identify factors that can influence the results. Including such variables could improve the accuracy and robustness of the predictions.

## Ethics statements

The data utilized in this study were sourced from the official website of the DKI Jakarta Environment Agency (https://lingkunganhidup.jakarta.go.id/). The dataset comprises daily air pollution standard index records spanning from January 2021 to August 31, 2024.

## CRediT authorship contribution statement

**Nurtiti Sunusi:** Conceptualization, Methodology, Software, Writing – original draft, Visualization. **Ankaz As Sikib:** Conceptualization, Methodology, Writing – review & editing, Validation, Supervision. **Sumanta Pasari:** Conceptualization, Methodology, Writing – review & editing.

## Declaration of competing interest

The authors declare that they have no known competing financial interests or personal relationships that could have appeared to influence the work reported in this paper.

## Data Availability

Data will be made available on request.
